# Certum Est, Quia Impossibile Est.—*Tertullian*

**Published:** 1920-05-29

**Authors:** 


### A FORTY-EIGHT HOURS' WEEK.

### Certum est, quia impossibile est.—*Tertullian.*

Concerning a forty-eight-hours' week for nurses, there are two schools—convinced and determined believers, and the "impossibles." Those who entered into the spirit of the war have almost, if not altogether, expunged the word "impossible" from their vocabularies. Empires invincible and invulnerable have crumbled into dust, and the world's outlook is changed. At the moment we are in the transition stage. The pendulum has swung, and, whereas it used to be all work and no play, now a vast multitude are singing "All play and no work."

Some day we shall arrive at a normal condition. But the old days are dead. There are no longer hereditary bond-slaves in Britain. By common consent the people have arrived at the conclusion that forty-eight hours is a fair week's work. This, so far as I am concerned, is not argument. I am simply chronicling what I believe to be true. And, if I am right, the conclusion is irresistible that, sooner or later, the nursing profession must find itself affected. The probationer of the future will revolt against entering a profession which demands complete sacrifice. Sermons and homilies on life and duty avail not. The tide is flowing, and no modern Mrs. Partington can stop it with a broom.

At the same time, those who declare that a forty-eight hours' week is impossible have much to say for themselves. Hundreds of thousands of middle-class families are living up to their last shilling, and sickness, where nurses' services are necessary, comes like a blast from the desert. Take even a mild case of diphtheria. Effectual nursing is quite as necessary as the most skilled medical treatment. To burden a man of the middle class with the expense of three nurses is to beggar him. The problem is real, and, I may add, terrible. It demonstrates, does it not, how far off we are from civilisation in the high sense of the term? I remember some years ago reading an address by Lord Rosebery on the subject of riches. I cannot quote the text. But the burden of his message was that the only value that riches possess is that the owner can provide the best possible treatment for the sick. The most skilled physicians, a long rest, travel, dainties, and medicine. Lord Rosebery was right. Honour and glory are dead-sea fruit to men or women who have to pay the forfeit of the child of their heart of hearts. On the rich sickness imposes the minimum of hardship. To the poor it spells tragedy, misery, and sometimes ruin.

The problem presents itself—Must a poor man employ three nurses if a forty-eight-hour week becomes recognised? The people who say "impossible" have arrived just here, and here they remain. They cannot do so. The pressure from behind will force them on. The forty-eight-hour week is coming, and the world will have to adjust its arrangements accordingly.

I venture to suggest that the first essential is to organise nursing on new and up-to-date lines, or, in other words, so as to suit the demands and requirements of the public. We have at present Trained Nurses and nothing else. In America every Employment Bureau advertises Trained Nurses and Practical Nurses, the latter being women who know something of nursing, something of housekeeping, and who are willing to do both at a lower scale of remuneration than the fully qualified, whose duties are exclusively with the sick. Sickness varies in seriousness, and in many middle-class homes a practical nurse would be immensely valuable and distinctly economic. Trained Nurses could not possibly suffer by recognising and helping to organise a lower grade. On the contrary, if this were done on well-thought-out lines undesirables could be completely excluded from employment, ultimately if not immediately. A very large number of Trained Nurses were led to believe that when a Registration Act was passed the untrained would automatically find their occupation gone, as in the case of Midwives. I took a great deal of trouble to expose the fallacy, and now the truth is apparent. The organisation of the untrained would go a long way to solve the problem of the employer and to keep the profession of nursing exclusive.

Should this idea find support it appears to me that the College of Nursing should take the task in hand. Neither the voluble Trade Union Hyde Park orator nor the apostle of the "impossible" school will help very much. Nor will shutting one's eyes to the trend of modern public opinion avail aught. The thing to do, and to do without delay, is to face the problem with courage and with determination to find a remedy that will at once help the public and the nurse. IERNE.

